# Short-Term Effects of a Multidisciplinary Residential Rehabilitation Program on Perceived Risks, Confidence Toward Continuous Positive Airway Pressure Treatment, and Self-Efficacy in a Sample of Individuals Affected by Obstructive Sleep Apnea Syndrome

**DOI:** 10.3389/fpsyg.2021.703089

**Published:** 2021-08-18

**Authors:** Federica Scarpina, Ilaria Bastoni, Simone Cappelli, Lorenzo Priano, Emanuela Giacomotti, Gianluca Castelnuovo, Enrico Molinari, Ilaria Maria Angela Tovaglieri, Mauro Cornacchia, Paolo Fanari, Alessandro Mauro

**Affiliations:** ^1^Istituto Auxologico Italiano, IRCCS, U.O. di Neurologia e Neuroriabilitazione, Ospedale S. Giuseppe, Piancavallo, Italy; ^2^“Rita Levi Montalcini” Department of Neurosciences, University of Turin, Turin, Italy; ^3^Istituto Auxologico Italiano, IRCCS, Laboratorio di Psicologia, Ospedale S. Giuseppe, Piancavallo, Italy; ^4^Psychology Department, Università Cattolica del Sacro Cuore, Milan, Italy; ^5^Istituto Auxologico Italiano, IRCCS, U.O. di Riabilitazione Pneumologica, Ospedale S. Giuseppe, Piancavallo, Italy

**Keywords:** OSA syndrome, CPAP therapy, obesity, perceived risk, self-efficacy, temperament

## Abstract

Continuous positive airway pressure (CPAP) therapy is the standard treatment for obstructive sleep apnea (OSA) syndrome. However, optimizing adherence to CPAP therapy of individuals remains very challenging for clinicians because of the role played by the psychological components. In this study, we verified the changes in cognitions and beliefs of individuals after a four-week multidisciplinary residential rehabilitation program targeting the adaptation to CPAP therapy for OSA syndrome. We assessed the components of perceived risks, confidence toward the treatment, and self-efficacy through the self-report questionnaire, namely the Self-Efficacy Measure for Sleep Apnea (SEMSA) questionnaire. We also explored the role played by the temperamental traits on the changes registered in these components after the treatment. Forty-five participants completed the rehabilitation program, showing a higher level of adherence to the treatment. Significant changes were observed in terms of confidence toward the treatment, although no change was reported in terms of perceived risks and self-efficacy. Moreover, those individuals with a higher persistent temperamental trait reported a significant improvement in perceived risks, in the absence of other significant results. After the rehabilitation treatment, our participants were more prone to consider the effect of CPAP treatment on health outcomes. This was in line with the educational aim of the rehabilitation treatment. The temperament seemed to play only a marginal role in the global changes reported by our participants. We discussed the need for behavioral interventions, in addition to education, in improving self-efficacy.

## Introduction

Continuous positive airway pressure (CPAP) therapy is the standard treatment for the obstructive sleep apnea (OSA) syndrome, a respiratory sleep disorder characterized by repeated episodes of partial or complete obstruction of the upper airway occurring during the inspiratory phase (American Academy of Sleep Medicine, 2014). The therapy consists of the use of a ventilator, which performs by blowing air into the throat *via* a mask, subtly increasing air pressure in the throat, and preventing the airway from narrowing. Despite its benefits, compliance of patients to CPAP therapy is not always satisfactory, and optimizing adherence of individuals to CPAP ventilator remains very challenging for clinicians. Dropout of patients is generally due to difficulties in using the ventilator and discomfort with the usage of the full-face mask. Moreover, it should be underlined that CPAP therapy requires a considerable alteration of the lifestyle and sleeping of an individual (Kribbs et al., 1993; Rosen et al., 2012). Crucially, non-adherence to the treatment emerges very early, within the first week of therapy (Weaver et al., 1997; Rosentha et al., 2000; Weaver and Grunstein, 2008). In fact, in this short time period, individuals develop personal cognitions and beliefs about CPAP therapy (i.e., outcome expectancies, risk perception, and self-efficacy), which shape the volition to use the ventilator (Aloia et al., 2005; Olsen et al., 2008; Weaver and Grunstein, 2008; Baron et al., 2011; Sawyer et al., 2011a,b) with the long-term effects on the level of adherence to treatment (Weaver and Grunstein, 2008; Sawyer et al., 2011a; Cayanan et al., 2019). Moreover, the subjective perception of the disease as well as its consequences on the health and psychological wellbeing of an individual do not necessarily reflect the objective severity of the illness and the need for treatment (Scarpina et al., 2020). This mismatch limits the efficacy of the treatment itself (Cayanan et al., 2019). Thus, the clinical procedures adopted in the earlier weeks to assist patients in their process of adaptation to ventilotherapy might play a significant role in increasing the long-term compliance of an individual to the use of CPAP.

In this study, we described an observational study with the aim to evaluate the short-term within-group effects of a four-week residential rehabilitation program in a sample of individuals affected by OSA syndrome. We focused on the changes in the cognitive components of perceived risks, confidence toward CPAP treatment, and self-efficacy, measured through the Self-Efficacy Measure for Sleep Apnea (SEMSA, Weaver et al., 2003) self-report questionnaire. The rehabilitation program was multidisciplinary: it targeted the adaptation to CPAP therapy for OSA syndrome (Budin et al., 2019); moreover, it regarded diet therapy, exercise training, and nutritional and psychological counseling.

Adherence to CPAP therapy is traditionally the main outcome of the process of adaptation, in which the psychological factors of individuals play a crucial role (Stepnowsky et al., 2002; Cayanan et al., 2019; Garbarino et al., 2020). In exploring which individual factors may enhance the subjective perception of positive outcomes associated with CPAP therapy in the case of OSA syndrome, in this study, we focused on the role of temperament. No previous study has been reported in the literature about the impact on the perceptions of individuals about CPAP therapy for OSA syndrome in the earlier weeks of treatment. Temperament is the inheritable and stable set of emotional and learning factors that underline the acquisition of the traits and attitudes of automatic emotional behavior of individuals (Cloninger et al., 1993; Cloninger, 1999). According to Maschauer et al. (2017), those individuals who show negative affectivity, social inhibition, and unhealthy lifestyle associated with reluctance to consult and/or follow medical advice (type D personality) have the long-term higher levels of non-compliance and poor treatment outcomes; conversely, individuals with a high internal locus of control and high self-efficacy, with self-refer for treatment, and having active coping skills are more likely to adhere to the CPAP treatment. However, in the previous literature, the role of temperament on the adherence to the treatment was verified as a long-term outcome (3 months and more after the beginning of the CPAP treatment), but not in short term (<3 months) (Maschauer et al., 2017). To provide the first evidence in this field, we explored if individual changes, specifically in terms of perceived risks, confidence toward CPAP treatment, and self-efficacy (Weaver et al., 2003) registered after a four-week residential program for OSA syndrome, might be related to the individual temperament.

## Methods

This observational study had a quasi-experimental pre–post design without the control group. This study was approved by the Ethics Committee of our Institution (Reference number: 21C924_2019). All participants were volunteers who gave informed written consent before participating in this study; they were free to withdraw at any time and were naïve to the rationale of this study. None of the participants were remunerated for their participation in this study; in fact, the National Sanitary System in Italy covers all hospital charges.

### Participants

We included in-patients consecutively recruited at their admission to the hospital between February 2019 and February 2020 and between November 2020 and April 2021. The recruitment was interrupted between February 2020 and November 2020 due to the COVID-19 pandemic worldwide.

Individuals were included in this study if: (i) they reported an Apnea–Hypopnea Index (AHI) higher than the value of 5 (Berry et al., 2012) at the full-night polysomnography (American Academy of Sleep Medicine, 2014) performed at their admission to our hospital, and (ii) they attended the rehabilitation program for the adaptation to CPAP therapy. We excluded individuals who used ventilotherapy in the past and who declared the regular use of hypnotic medications. Also, we excluded smokers; individuals with a history of alcohol abuse; and individuals with a history of cardiovascular, psychiatric, neurological disorders, or any concurrent medical condition that was not related to OSA syndrome.

Due to the negative side effects of OSA syndrome on cognition (Alomri et al., 2021; Legault et al., 2021), all participants performed a global assessment of the cognitive functioning through the Mini-Mental State Examination (Folstein et al., 1975; Italian version; Magni et al., 1996), the Clock-Drawing Test (Rouleau et al., 1992; Italian version: Siciliano et al., 2016), and the Frontal Assessment Battery (Dubois et al., 2000; Italian version: Appollonio et al., 2005).

Participants completed the Temperament and Character Inventory-Revised published by Martinotti et al. (2008). It is a 240-item self-administered questionnaire designed to assess four main temperamental traits as follows: (i) *novelty seeking*, associated with the emotion of anger, expresses the level of activation of exploratory activity; (ii) *harm avoidance*, related to fear, reflects the efficiency of the behavioral inhibition system; (iii) *reward dependence*, associated with attachment, refers to reward-based behavioral maintenance, and (iv) *persistence*, related to ambition, expresses the maintenance of behavior such as resistance to frustration. The questionnaire also measures three character dimensions, namely, self-directedness, cooperativeness, and self-transcendence, which denote self-concept and individual differences in goals and values and which influence choices and intentions.

### The Rehabilitation Program

Individuals took part in a residential four-week multidisciplinary approach targeting the adaptation to CPAP therapy for OSA syndrome and the improvement of quality of life (Budin et al., 2019). The process of adaption to the use of CPAP starts after the polysomnography, and it is supervised by trained nurses. The nurses assist patients in using autonomously the CPAP and in increasing the level of awareness about the benefits associated with the ventilotherapy when used during sleeping. Before the first night of CPAP therapy, the nurse informs the patient about the procedure that will be followed during the four weeks; moreover, she/he illustrates the CPAP, its components, and its functioning. Together with the patient, the nurse choices the most suitable mask, taking into account the facial shape of patients. While the patient wears the mask, he/she sees him/herself in a mirror to verify the right position of the mask on the face. In addition, the nurse verifies the level of comfort. Then, the patient experiences the use of CPAP for 15/20 min. Later, the CPAP is delivered to the patient, who can start using it during sleeping. During night, the nursing staff supervises patients in ventilotherapy, assisting them in case of necessity. For example, if the patient removes the mask during night, the nursing staff wakes up and invites him/her to replace it in the right position. The next morning, the nursing staff verifies if the CPAP has been used correctly during night through the CPAP output.

The rehabilitation program includes individual and group-based psychological and educational sessions. The individual sessions consist of psychological consultation once a week (overall, four sessions), in which specialized psychologists provide support for psychological distress, such as depression and anxiety problems. Group sessions are scheduled every hour once a week (four sessions overall) and provide impersonal education about psychosocial risk factors and promotion of healthy habits.

The program entails daily sessions (six days a week) of aerobic activity that included 30 min/day of recreational activities at low intensity (walking) and 45 min of physical activities at high intensity (Lanzi et al., 2015).

All participants receive nutritional counseling, in which dietary assessment, evaluation of nutrient intake and adequacy, nutritional status, anthropometric measures, eating patterns, and history of overweight are performed. Thus, a personalized diet (i.e., 50% of energy from carbohydrates, 30% from lipids, and 20% from proteins) is furnished to each patient, in which the caloric intake is set at approximately 80% of resting energy expenditure. Every week, the diet is checked and adapted (Panasini et al., 2015). Participants also receive hourly group impersonal nutritional counseling twice a week relative to healthy eating, general nutrition, and core food groups.

### Main Outcome: The SEMSA Questionnaire

At T0 (at the beginning of the treatment) and T1 (after 4 weeks, at the end of the rehabilitation program), participants filled out the Italian version (Manni and Palagini, 2016) of the SEMSA questionnaire (Weaver et al., 2003). This questionnaire assesses the following: (i) risk perception—the perceived vulnerability of patients to health risks (i.e., that untreated OSA syndrome would result in a negative outcome); (ii) outcome expectancy—perceived expectations regarding the potential of the behavior to reduce those risks (i.e., the perception that the use of CPAP would result in positive consequences in the life of patients); and (iii) treatment self-efficacy—perceived ability to perform the behavior (i.e., the perception that the patient has the wherewithal to use the CPAP effectively under a wide range of circumstances). The internal consistency coefficient of the total scale was 0.92 with item-to-total correlations ranging from 0.26 to 0.66 (Manni and Palagini, 2016). The Cronbach's α statistic for each of the three subscales was >0.85. Test–retest reliability coefficients (*N* = 20) were 0.68, *p* = 0.001 for perceived risk; 0.77, *p* < 0.0001 for outcome expectancies; and 0.71, *p* = 0.0005 for the treatment self-efficacy subscales.

### Secondary Outcomes

All the following measures were collected twice, at T0 and T1. We assessed the effect of rehabilitation treatment on the psychological wellbeing of participants through the Italian version (Grossi et al., 2006) of the Psychological General Well Being Index (PGWBI) (Dupuy, 1984). The questionnaire measures the domains of anxiety, depressed mood, positive wellbeing, self-control, general health, and vitality. We also measured the subjective perception of sleeping efficacy through three questionnaires: the Epworth Sleepiness Scale (Johns, 1991; Italian version: Vignatelli et al., 2003), which focuses on the pathological daily sleepiness (the higher the score the worse the sleepiness); the Pittsburgh Sleep Quality Index (Buysse et al., 2005; Italian version: Curcio et al., 2013), which assesses the sleep quality (the higher the score, the worse the quality); and the Stanford Sleepiness Scale (Hoddes, 1972), which quantifies the subjective level of alertness throughout the day (the higher the score the better the level of alertness).

To verify the effect of rehabilitation treatment on the attentional abilities, our participants performed the Flanker's test from the Psychology Experiment Building Language (PEBL)—Psychological Test Battery (Mueller and Piper, 2014) and as described in the study by Scarpina et al. (2020). This computerized attentional test measures the response inhibition. Participants were asked to detect visual stimuli (i.e., the targets) presented centrally while ignoring stimuli (i.e., the flankers) presented spatially close to them. The flankers correspond either to the same directional (right vs. left) response as the target (congruent condition), to the opposite response (incongruent condition), or to neither (neutral condition). There is also an experimental condition in which no flanker is shown. The flankers affect the behavioral responses to the central target positively (increasing the level of accuracy and the detection velocity), when they are consistent with the target response, or negatively (decreasing the level of accuracy and the detection velocity), when they are incompatible with the target. Overall, in the version used in this study, 160 trials were tested, 40 for each condition. Before the task, participants performed eight run-in trials to familiarize themselves with it. Further technical details can be found online (http://pebl.sourceforge.net) and in the study by Mueller and Piper (2014). For each trial, we registered the reaction time in milliseconds and the level of accuracy expressed in percentage (Scarpina et al., 2020).

Finally, after the four-week adaptation program, the standard full-night polysomnography was performed and compared with the measurement performed during admission.

**Figure 1:**
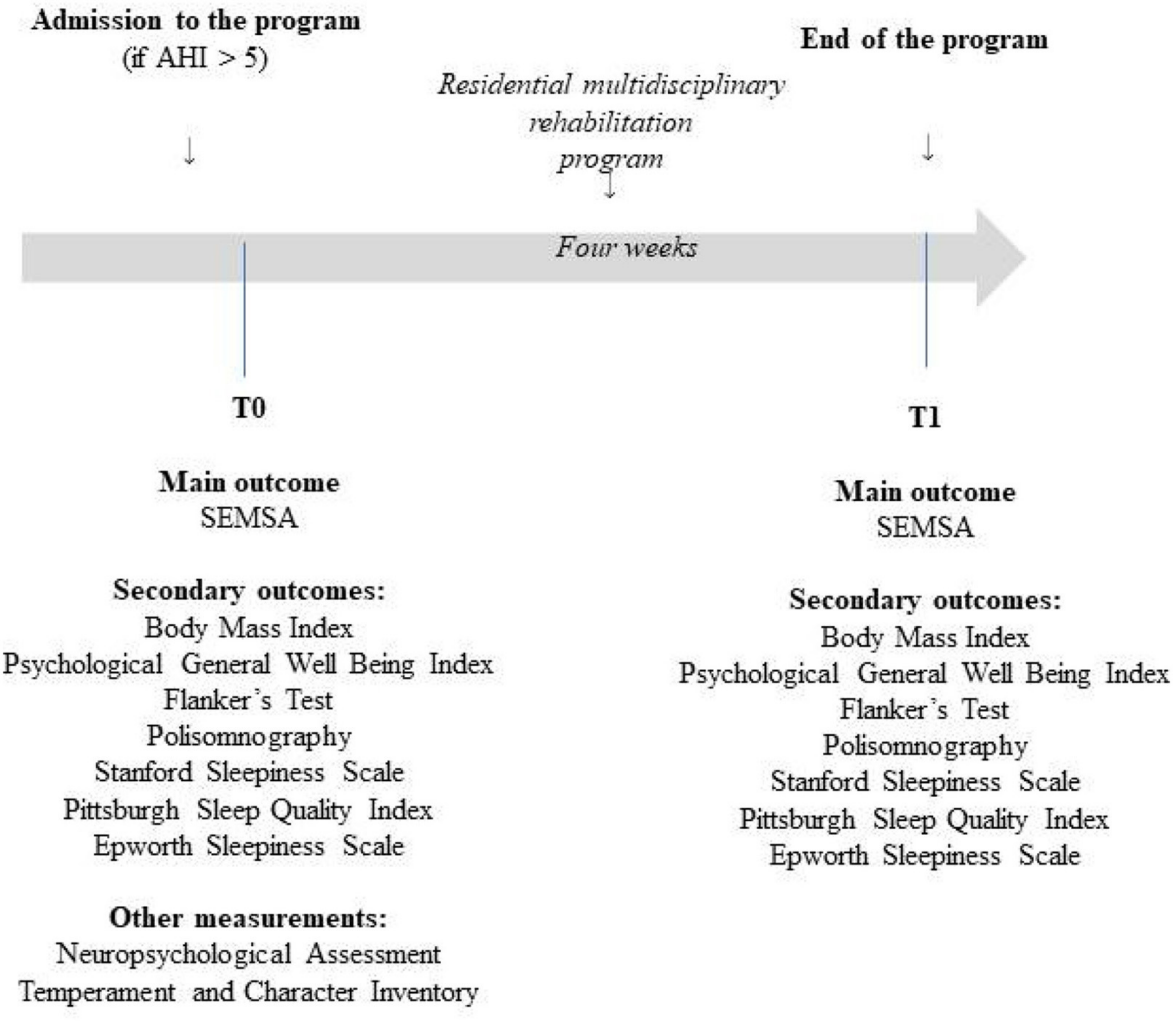
Schematic representation of the procedure.

The timeline of our study is shown in Figure 1.

## Analyses

### Adherence to CPAP

Adherence to CPAP was measured in terms of the number of nights of use and number of hours of use per night (Bravata et al., 2012). Adherence was classified into the following three groups: “good” if the usage was at least four hours per night for at least 70% of nights; “some” if the patient had used the device at least 10% of nights but less than the threshold for “good;” and “none/poor” if the patient used the device <10% of nights (Miech et al., 2019).

### Primary Outcome

We investigated the changes in the three components measured through the SEMSA questionnaire, comparing the scores reported at the baseline (T0) and after the treatment (T1) within our sample using the Wilcoxon signed-rank test. Successively, we investigated if the changes observed between the baseline (T0) and after the four-week rehabilitation program (T1) in the SEMSA questionnaire scores were explained by the level of OSA syndrome severity and level of obesity of participants registered at their admission to the rehabilitation (T0) using the linear regression analysis (Myers et al., 2012). In detail, for each component of SEMSA, we computed the difference between the score reported at T0 and the score reported at T1 (i.e., Δ score). The Δ scores were used in the linear regression model, in which the AHI, indicating the level of OSA syndrome severity, and the body mass index (BMI), as the index of obesity severity, were used as statistical predictors. We reported the *R*-squared as a goodness-of-fit measure. We also evaluated the significance of the model through *F*-value and *p*-value. The relative contribution of the two predictors (i.e., AHI and BMI) with the independent variable (i.e., Δ SEMSA score for each component of the questionnaire) was analyzed. The variance inflation factor (VIF) was used as a measure of multicollinearity.

### Secondary Outcomes

We investigated the changes associated with the four-week treatment in the PGWBI questionnaire and the three questionnaires assessing the subjective sleeping quality, comparing the scores reported at the baseline (T0) and after the treatment (T1) within our sample, using the Wilcoxon signed-rank test. We used the related-sample *t*-test to assess the changes in the BMI and the objective measurements of sleeping from polysomnography. To explore the changes in attentional abilities measured using the Flanker's test after the four-week treatment, we performed a repeated measure ANOVA with the factor *Condition* (i.e., neutral, congruent, incongruent, and single) and the factor *Time* (i.e., T0 vs. T1), independently for the reaction time in milliseconds and the level of accuracy expressed in percentage, as done in the study by Scarpina et al. (2020).

### The Role of Temperament

We compared the scores of our participants reported at the Temperament and Character Inventory-Revised with those reported by the normative sample (Martinotti et al., 2008). Successively, the scores were converted into *T*-scores (which have a normal distribution with a mean of 50 and an SD of 10) as done in the study by Cloninger et al. (1994). We aimed to verify if changes observed between the baseline (T0) and after the four-week rehabilitation program (T1) in the scores relative to the SEMSA questionnaire were explained by the temperamental traits of participants. To respond to this aim, we used a linear regression analysis (Myers et al., 2012), as previously done. Specifically, we computed the difference between the score reported at T0 and the score reported at T1 (i.e., Δ score). Successively, we analyzed the correlation and directionality of the data to formulate the statistical model as follows: the Spearman's correlation coefficient was computed between the Δ scores relative to the SEMSA subscales and the *T*-scores relative to the four temperamental traits from the Temperament and Character Inventory-Revised. Those Δ scores that were significantly associated with one or more *T*-scores of the temperamental traits (*p* ≤ 0.05) were used in the linear regression model. We reported the *R*-squared as a goodness-of-fit measure, the significance of the model through *F*-value and *p*-value, the relative contribution of the temperamental trait(s) included in the statistical model with the independent variable (i.e., Δ SEMSA scores), and the VIF as the measure of multicollinearity.

## Results

Fifty-two individuals (i.e., 32 women; 20 men; mean age in years = 54.61; SD = 9.52; range = 34–75; mean education in years = 11.88; SD = 3.82; range = 5–23) were enrolled. However, only 45 individuals (i.e., 27 women and 18 men) were included in this study, since 7 participants did not complete the rehabilitation program. The mean age in years was 53.6 (SD = 9.85; min–max = 34–72); the mean education in years was 12.26 (SD = 3.97; min–max = 5–23). At their admission to the rehabilitation program, 80% of our participants reported a severe apnea, with the AHI higher than 30, 4.44% of our participants reported a mild sleep apnea (5 ≤ AHI < 15), and 15.55% of our participants reported a moderate apnea (15 ≤ AHI < 30). Moreover, 91.11% of participants suffered from class III (high risk) obesity, since the BMI was equal to or >40, 6.66% of participants suffered from class II (moderate risk; BMI between 35.0 and 39), and 2.22% of participants suffered from class 1 (low risk; BMI between 30.0 and 34.9). The observed pattern was in line with the observation that OSA syndrome occurs frequently in obesity, as well as obesity is a high-risk factor for the development and progression of sleep apnea (Schwartz et al., 2008). The mean score relative to the Mini-Mental State Examination (Folstein et al., 1975) was 28.84 (SD = 1.06; min–max = 26–30), the Clock-Drawing Test (Rouleau et al., 1992) was 8.94 (SD = 1.73; min–max = 0–10), and the Frontal Assessment Battery (Dubois et al., 2000) was 16.95 (SD = 1.16; min–max = 12–18).

### Adherence to CPAP

When we assessed the level of adherence to CPAP therapy, the majority of our participants (94.87%) had good use of CPAP after the four-week rehabilitation treatment, although only 5.4% of participants reported some good use of CPAP. The mean number of night of CPAP used was 24.91 (SD = 3.09; range 15–29), and the mean time of CPAP was 6 h and 4 min for night (SD = 0.05; range 3 h and 26 min−9 h and 3 min).

### Main Outcome

The scores registered at T0 and T1 as well as the statistical results for the three components investigated through the SEMSA questionnaire are reported in Table 1.

**Table 1:** Mean, SD, and minimum (min) and maximum (max) for each scale relative to the Self-Efficacy Measure for Sleep Apnea (SEMSA) questionnaire registered at T0 (i.e., before the treatment) and T1 (i.e., after the four-week rehabilitation program).

	**T0**	**T1**	**Statistical results**
Perceived Risk (range score 1–4)	M = 1.91 SD = 1.24 min-max = 0–4	M = 2.28 SD = 1.47 min-max = 0–4	W (44) = 542; p = 0.07; d' = 0.27
Outcome Expectancies (range score 1–4)	M = 2.43 SD = 0.93 min-max = 0–4	M = 2.78 SD = 0.96 min-max = 0–4	W(44) = 597; **p = 0.004**; d' = 0.37
Treatment Self-Efficacy (range score 1–4)	M = 1.28 SD = 0.79 min-max = 0–3.11	M = 1.14 SD = 0.81 min-max = 0–3.7	W(44) = 249; p = 0.78; d' = 0.17

*We reported statistical difference, and the significant difference between T0 and T1 scores is given in bold. For each scale, we reported the range score, according to the seminal article*.

We observed that the level of individual perceived vulnerability to health risks and the level of perceived self-efficacy did not change after the rehabilitation; instead, our participants reported higher perceived expectations about the positive outcome of CPAP therapy on quality of life after the treatment. We successively verified the predictive role of the level of OSA syndrome severity (i.e., AHI) and the level of obesity (i.e., BMI) registered during admission on the changes in the SEMSA questionnaire scores. The VIF value relative to the AHI was 1.047; for the BMI, it was 1.047. Neither the AHI [β = −0.005, *t*_(41)_ = −0.83, *p* = 0.5] nor the BMI [β = 0.03, *t*_(41)_ = 1.08, *p* = 0.28] predicted the changes in the perceived risk score [*R*^2^ = 0.03; *F*_(1, 41)_ = 0.78; *p* = 0.64]. Changes in the outcome expectancies score were not predicted by AHI [β = −0.004, *t*_(41)_ = −0.8, *p* = 042] or by BMI [β = 0.002, *t*_(41)_ = 0.1, *p* = 0.91] [*R*^2^ = 0.01; *F*_(1, 41)_ = 0.31; *p* = 0.72]. Similarly, no effect was registered relative to the AHI [β = −0.007, *t*_(41)_ = −0.8, *p* = 0.42] and the BMI [β = −0.07, *t*_(41)_ = −1.91, *p* = 0.06] [*R*^2^ = 0.11; *F*_(1, 41)_ = 2.59; *p* = 0.08] when we focused on the treatment self-efficacy score. These results suggested that the level of obesity as well as the level of OSA syndrome severity did not explain the changes registered in the SEMSA scores after the rehabilitation treatment.

### Secondary Outcomes

Mean, SD, and range registered at T0 and T1 for all the assessed factors (except for the Flanker's test) are reported in Table 2.

**Table 2:** Mean, SD, and minimum (min) and maximum (max) for the secondary outcomes measured in this study at T0 (i.e., before the treatment) and T1 (i.e., after the four-week rehabilitation program).

	**T0**	**T1**	**Statistical results**
Body mass index	M = 46.69 SD = 7.08 min-max = 33.35–69.18	M = 44.59 SD = 6.65 min-max = 31.34–65.66	t(44) = 10.84; **p = 0.003**; d' = 0.3
**Psychological well-being: Psychological General Well Being Index**			
Anxiety (range score 0–25)	M = 16.11 SD = 4.66 min-max = 5–24	M = 20.93 SD = 3.76 min-max = 8–25	W(45) = 960.5; **p < 0.001**; d' = 1.13
Depression (range score 0–15)	M = 11.86 SD = 2.65 min-max = 2–15	M = 13.64 SD = 1.69 min-max = 8–15	W(45) = 587; **p < 0.001**; d' = 0.8
Positive well-being (range score 0–20)	M = 10.24 SD = 4.33 min-max = 3–19	M = 14.62 SD = 3.73 min-max = 6–20	W(45) = 952; **p < 0.001**; d' = 1.84
Self-control (range score 0–15)	M = 10.66 SD = 5.99 min-max = 3–15	M = 12.95 SD = 2.44 min-max = 6–15	W(45) = 622.5; ***p*** **< 0.001**; d' = 0.5
General healthy (range score 0–15)	M = 8.11 SD = 3.07 min-max = 3–13	M = 10.66 SD = 2.73 min-max = 5–15	W(45) = 669; **p < 0.001**; d' = 0.87
Vitality (range score 0–20)	M = 9.62 SD = 4.8 min-max = 1–20	M = 15.13 SD = 4.15 min-max = 0–20	W(45) = 869; **p < 0.001**; d' = 1.22
Total score (range score 0–110)	M = 66.62 SD = 19.03 min-max = 28–105	M = 87.95 SD = 15.14 min-max = 53–110	W(45) = 1024; **p < 0.001**; d' = 1.22
**Sleep—quantitative measurement: polysomnography**			
Number of apnea/hypopnea events per hour of sleep (Apnea/Hypopnea Index—AHI)	M = 59.84 SD = 34.91 min-max = 11–150.5	M = 2.85 SD = 2.88 min-max= 0.1–15.4	t(38) = 10.53; **p < 0.001;** d' = 2.3
Number of hypopnea events per hour of sleep (Hypopnea Index—HI)	M = 44.74 SD = 35.65 min-max = 0.8–127.8	M = 2.53 SD = 2.66 min-max = 0.1–14	t(38) = 7.73 **p < 0.001;** d' = 1.67
Number of apnea events per hour of sleep (Apnea Index—AI)	M = 29.43 SD = 20.78 min-max = 0.4–87.8	M = 0.35 SD = 0.62 min-max = 0–2.5	t(38) = 8.62; **p < 0.001;** d' = 1.97
Number of blood oxygen desaturation events per hour of sleep	M = 54.48 SD = 30.79 min-max = 10.9–136.5	M = 2.27 SD = 2.25 min-max = 0-9.4	t(38) = 10.64; **p = 0.019;** d' = 2.39
Time with SaO_2_ <90% (% of total sleep time)	M = 65.81 SD = 81.15 min-max = 3.5–100	M = 8.87 SD = 21.62 min-max = 0–100	t(38) = 4.03; **p < 0.001;** d' = 0.95
Average minimum SaO_2_ during desaturations (%)	M = 82.42 SD = 4.82 min-max = 69 −89	M = 88.27 SD = 14.45 min-max = 0–95	t(38) = 2.45; **p < 0.001;** d' = 0.54
Minimum oxygen saturation (%)	M = 65.82 SD = 10.28 min-max = 49–87.4	M = 86.8 SD = 3.56 min-max = 80–95	t(38) = 13.58; **p < 0.001;** d' = 2.27
Average sleeping time in hour	M = 7.26 SD = 0.74 min-max = 4–8	M = 6.24 SD = 0.74 min-max = 5.1–8.1	t(38) = 6.87; **p < 0.001;** d' = 1.37
**Sleep—subjective evaluation**			
Stanford sleepiness scale (range score 0–7)	M = 2.84 SD = 1.06 min-max = 1.11–5.33	M = 1.96 SD = 0.63 min-max = 1.05–3.94	W(45) = 82; **p < 0.001;** d' = 1
Pittsburgh sleep quality index (range score 0–21)	M = 7 SD = 3.14 min-max = 1–13	M = 4.97 SD = 2.61 min-max = 1–13	W(45) = 165; **p < 0.001;** d' = 0.7
Epworth sleepiness scale (range score 0–24)	M = 8.82 SD = 5.27 min-max = 0–22	M = 3.73 SD = 3.5 min-max = 0–12	W(45) = 24; **p < 0.001;** d' = 1.13

*We reported statistical results, and the significant difference between T0 and T1 scores is given in bold*.

After the four-week program, participants reported a significantly lower BMI in comparison with the baseline. They also reported an overall increase in the psychological wellbeing, as shown by the results relative to the PGWBI scores. Considering the subjective sleeping quality, participants reported a significantly reduced level of daily sleepiness, measured through the Epworth Sleepiness Scale (Johns, 1991), as well as they reported a significantly higher level of alertness throughout the day (Stanford Sleepiness Scale, Hoddes, 1972) and a higher level of sleep quality (Pittsburgh Sleep Quality Index, Buysse et al., 2005). Finally, in all indexes measured through the polysomnography, we registered a significant improvement.

**Figure 2:**
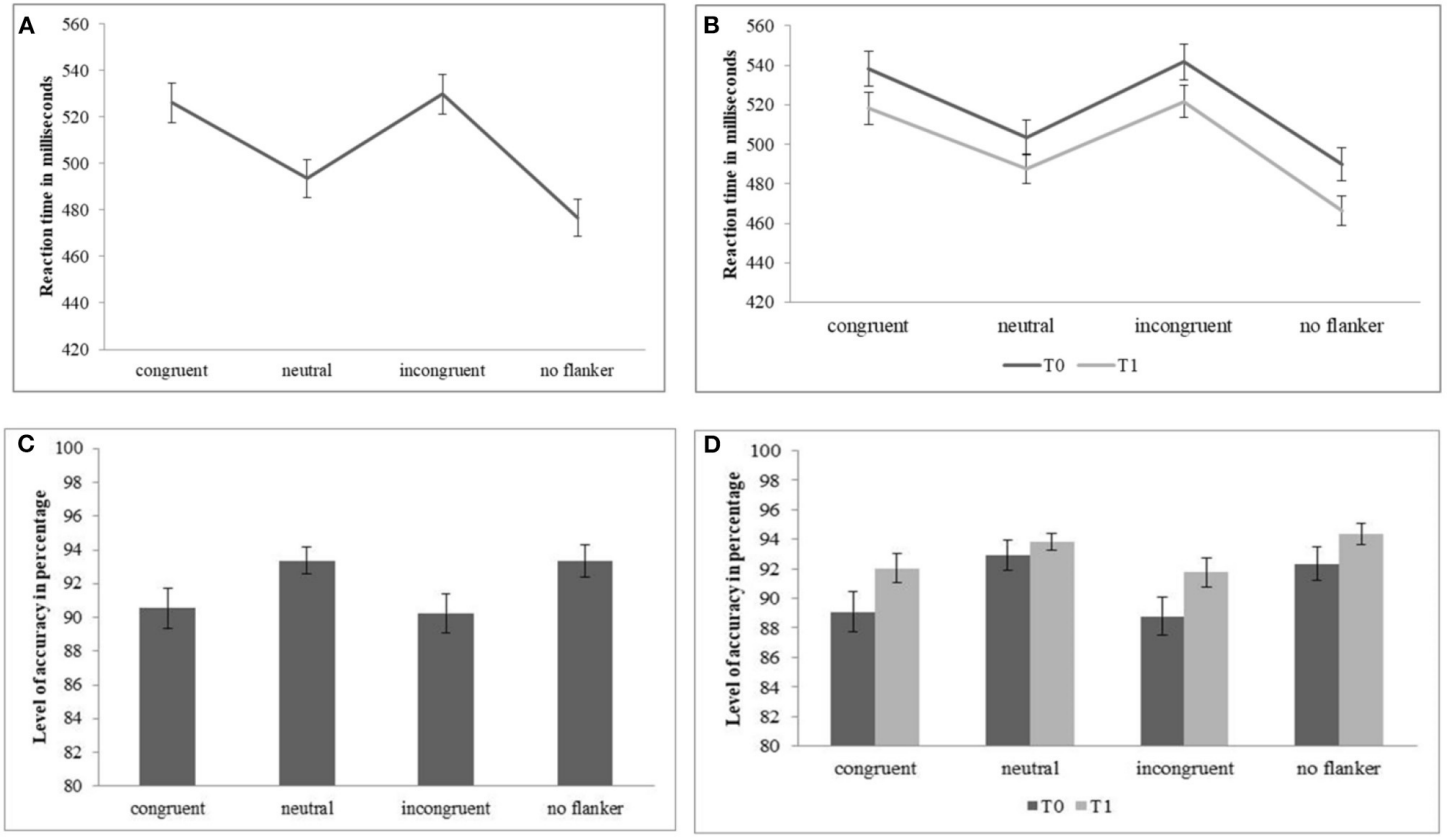
Mean and SD (vertical lines) relative to the reaction time in milliseconds and the level of accuracy in percentage registered at the Flanker's test. Specifically, in panels **(A,C)**, the data in relation to the four experimental conditions are shown; in panels **(B,D)**, the data in relation to the four experimental conditions split for the two measurements (T0 vs. T1) are shown.

We used the Flanker's test to verify the changes in attentional abilities in our sample. When we considered the reaction time, we observed the significant main effect of *Condition* [*F*_(3, 126)_ = 259.132; *p* < 0.001; partial eta-squared *p* = 0.86]; according to the *post hoc* estimated marginal means comparisons Bonferroni-corrected, the presence of the incongruent or congruent flanker affected the detection velocity in comparison with the conditions in which the flanker was not shown or it was neutral; all the comparisons were significant [*p* < 0.001], except between the congruent and the incongruent conditions [*p* = 0.35] (Figure 2A). Crucially, we observed the significant main effect of *Time* [*F*_(1, 42)_ = 19.07; *p* < 0.001; partial eta-squared *p* = 0.31], in which participants showed significant lower reaction time in T1 (*M* = 496; SD = 71) in comparison with T0 (*M* = 514; SD = 73). Finally, the interaction *Condition* × *Time* was not significant *F*_(3, 126)_ = 2.21; *p* = 0.09; partial eta-squared *p* < 0.001] (Figure 2B). When we considered the level of accuracy, we observed the significant main effect of *Condition* [*F*_(3, 126_) = 15.34; *p* < 0.001; partial eta-squared *p* = 0.26]; according to the *post hoc* estimated marginal means comparisons Bonferroni-corrected, the presence of an incongruent or congruent flanker affected the level of accuracy in comparison with the conditions in which the flanker was not shown or it was neutral; all the comparisons were significant (*p* < 0.001), except between the congruent and the incongruent conditions (*p* = 1) and the neutral and no flanker conditions (*p* = 1) (Figure 2C). No significant main effect of *Time* [*F*_(1, 42)_ = 3.34; *p* = 0.07; partial eta-squared *p* = 0.07] emerged, where the level of accuracy observed at T1 (*M* = 92.99; SD = 7.48) and T0 (*M* = 90.77; SD = 8.21) overlapped. Finally, the interaction *Condition* × *Time* was not significant [*F*_(3, 126)_ = 1.38; *p* = 0.25; partial eta-squared *p* = 0.03] (Figure 2D). This pattern of results suggested that the performance of our participants was in agreement with the Flanker's effect, mirroring the results reported by Scarpina et al. (2021). Crucially, after the rehabilitation, participants were faster in detecting the visual targets, suggesting an improvement of their attentional abilities. No significant change emerged in the level of accuracy; however, it should be considered that the percentage of accuracy was very high, suggesting a ceiling effect in our data.

### The Role of Temperament

**Figure 3:**
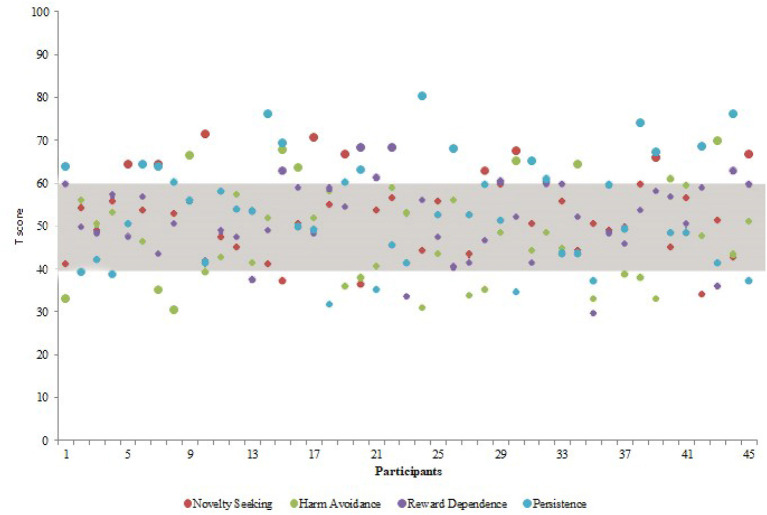
*T*-score (*y*-axis) relative to each temperamental trait (novelty seeking in red, harm avoidance in green, reward dependence in purple, and persistent in green) measured through the Temperament and Character Inventory-Revised for each participants (*x*-axis). The gray area represents the medium range according to Martinotti et al. (2008).

As shown in Table 3, our participants showed significantly higher scores in the novelty seeking and persistent temperamental traits, when compared with the normative data, with no other significant difference. The *T*-score of each participant at the four temperamental traits is shown in Figure 3.

**Table 3:** Mean, SD, and minimum (min) and maximum (max) for each scale of the Temperament and Character Inventory-Revised by Martinotti et al. (2008).

	**M**	**SD**	**min-max**	**Normative data**		**Statistical comparison**
				**M**	**SD**	
Novelty seeking (range score 0–175)	102.31	12.09	78–126	98.5	12.9	t_(50)_ = 2.04 ***p*** **= 0.04** d'= 0.3
Harm avoidance (range score 0–165)	93.75	16.06	68–125	96.4	14.4	t_(48)_ = 1.08 *p* = 0.28; d' = 0.17;
Reward dependence (range score 0–150)	103.53	11.57	75–125	101.4	13	t_(50)_ = 1.19 *p* = 0.23; d' = 0.17
Persistence (range score 0–175)	121.93	18.01	90–160	116.3	14.4	t_(47)_ = 2.05; ***p*** **= 0.04;** d' = 0.34;

*We reported mean and SD relative to the Italian normative sample (N = 740; Martinotti et al., 2008) as well as the statistical comparison; bold values denote the significant results. For each scale, we reported the range score, according to the seminal article*.

We observed the following scores relative to the three character dimensions as follows: self-directedness (*M* = 82.92, SD = 49.12, and range = 25–176); cooperativeness (*M* = 93.25, SD = 37.46, and range = 36–153); and self-transcendence (*M* = 71.27, SD = 14.38, and range = 39–97).

In Table 4, we reported the Spearman's correlation coefficient computed between the *T*-scores relative to the four temperamental traits from the Temperament and Character Inventory and the Δ scores (i.e., the changes between T0 and T1) relative to three components measured through the SEMSA questionnaire.

**Table 4:** Spearman's correlation *(*ρ score and *p* value) between the Δ score (i.e., the difference between T1 and T0 scores) relative to the SEMSA questionnaire scores and the *T*-scores for the temperamental traits, measured through the Temperament and Character Inventory-Revised.

	**SEMSA questionnaire**		
	**Perceived risk**	**Outcome expectancies**	**Treatment self-Efficacy**
Novelty seeking	ρ = −0.06 *p* = 0.66	ρ = 0.04 *p* = 0.75	ρ = 0.05 *p* = 0.74
Harm avoidance	ρ = −0.11 *p* = 0.45	ρ = 0.04 *p* = 0.77	ρ = 0.12 *p* = 0.42
Reward dependence	ρ = 0.11 *p* = 0.94	ρ = 0.16 *p* = 0.28	ρ = 0.14 *p* = 0.35
Persistent	**ρ = 0.29** ***p*** **= 0.050**	ρ = −0.01 *p* = 0.94	ρ = −0.29 *p* = 0.055

*N = 45*.

*Bold values denote the significant results*.

A significant positive relationship emerged relative to the changes observed in the perceived risk and the persistent temperamental trait, with no other significant results (Table 4). According to the linear regression model, the persistent temperamental trait significantly predicted the changes in the perceived risk score [β = 0.03, *t*_(44)_ = 2.02, *p* = 0.05; VIF = 1] [*R*^2^ = 0.29; *F*_(1, 43)_ = 4.09; *p* = 0.05].

## Discussion

In this study, we aimed to verify the changes in the cognitive components of risk perception, outcome expectancy, and self-efficacy relative to CPAP therapy (SEMSA, Weaver et al., 2003) after a multidisciplinary residential four-week rehabilitation program, in individuals affected by OSA syndrome. After our intervention, individuals reported significantly higher perceived expectations about the positive outcome of CPAP therapy on quality of life. However, no change was observed in the other two components measured by the questionnaire, that is, the level of perceived vulnerability of patients to health risks (i.e., risk perception) and the perceived level of self-efficacy. These results were not explained by the level of obesity as well as the level of OSA syndrome severity during admission to the rehabilitation treatment. Thus, after the rehabilitation treatment, our participants were more prone to consider the effect of CPAP therapy on health outcome, meaning how much they expected that the CPAP therapy potentially decreased the health risks and increased their quality of life (i.e., outcome expectancies) (Weaver et al., 2003). This result was strictly related to the aim of our rehabilitation treatment, which was to provide participants with detailed information about CPAP therapy. For its nature, this intervention could be defined as informative and educational. However, because of this peculiar characteristic, it might not be entirely efficient in changing the level of self-efficacy after four weeks in which individuals were involved in a residential program. In fact, the most powerful source of self-efficacy information is the personal experience of individuals (Bandura, 1997). In this clinical context, Weaver et al. (2003) defined self-efficacy as the perception that the individual has about the wherewithal to use the ventilator effectively under a wide range of daily life circumstances. However, during our rehabilitation, participants were highly supported by clinicians, perhaps with an effect on the perception of self-experience of being actively and independently involved in the use of CPAP. We also observed no changes in the risk perception, meaning how much our participants perceived themselves as vulnerable to health risks and how much they were aware of the negative outcome associated with untreated OSA syndrome (Weaver et al., 2003). This result was surprising since individual and in-group activities proposed in our rehabilitation program were designed to decrease health-risk behaviors, such as eating habits and physical activities. This represents a very crucial point; in fact, if participants do not perceive the role of some negative behaviors in enhancing the OSA syndrome, they would not avoid them. Once again, the fact of being in a residential program, in which all participants were involved in activities chosen by the multidisciplinary team, might play a considerable role in the perception of the effect of activities on the health outcomes.

Interestingly, after our rehabilitation intervention, the level of adherence to CPAP was very high in our group, and the changes were observed in all the rehabilitation outcomes. According to the polysomnographic indexes, the objective sleep quality increased after the treatment; also, the sleep quality was subjectively perceived as higher (i.e., Pittsburgh Sleep Quality Index) (Buysse et al., 2005 Italian version: Curcio et al., 2013); our participants reported reduced sleepiness (i.e., Epworth Sleepiness Scale) (Johns, 1991; Vignatelli et al., 2003) and conversely, higher levels of alertness (i.e., Stanford Sleepiness Scale) (Hoddes, 1972) throughout the day. These results were in agreement with the decreased reaction time observed in the Flanker's test from the PEBL—Psychological Test Battery (Mueller and Piper, 2014; Scarpina et al., 2020) after the rehabilitation, and our participants were faster in recognizing the visual stimuli, suggesting an amelioration of their attentional abilities. Overall, participants described a better global quality of life (i.e., PGWBI, Dupuy, 1984). Thus, overall, our rehabilitation approach showed the potential to change several health components in our participants. Nevertheless, it seemed to be the only partially efficient in changing the cognitions of participants. Weaver (2019) recently underlined the importance to adopt behavioral interventions (i.e., cognitive behavioral therapy and motivational enhancement therapy), in addition to education, improving the adherence and the self-efficacy of patients, as reported in some previous studies (Aloia et al., 2001, 2013; Richards et al., 2007; Stepnowsky et al., 2007; Weaver and Grunstein, 2008; Shannon et al., 2017). Moreover, considering the role played by vicarious experience or modeling in shaping self-efficacy (Bandura, 1997), group activities in which information can be obtained through the performance and experience of others might be adopted. Moreover, in assigning participants to the groups, the clinicians could not strictly take into account the level of obesity and OSA disease severity, since in our sample, both components did not play a significant role in changes registered after the treatment.

In this study, we also verified the role of the temperamental traits (Cloninger et al., 1993, 1994; Cloninger, 1999) on the cognitive components measured through the SEMSA questionnaire (Weaver et al., 2003). To the best of our knowledge, no previous study investigated this relationship in the case of short-term outcomes registered after rehabilitation programs. Those individuals with a higher persistent temperamental trait reported a significant improvement in perceived risks, although the other temperamental traits were not implicated in the changes of individuals. According to Cloninger et al. (2012), individuals with a higher expression of the persistent temperamental trait can be described as determined and ambitious; moreover, they tend to persevere in their behaviors despite fatigue or frustration, even though in the absence of an immediate reward. In the clinical setting, those individuals who are highly persistent seem to be more prone to pro-health activities. In addition, a high level of persistence plays a protective effect on emotional functioning, reducing negative emotions and increasing positive ones (Cloninger et al., 2012). In this study, we observed that those individuals with a persistent temperamental trait were more prone to link their commitment to CPAP therapy to health-positive outcomes, even though the difficulties they might experience in the adaptation process. However, solely changes in risk perception, but not in the other cognitive components of outcome expectancies and the treatment self-efficacy, were related to the persistent temperamental trait in this study. It might be hypothesized that the role of temperament was not entirely crucial in shaping outcome expectancies because of the informative and educational nature of the proposed intervention. Instead, the absence of any effect of temperament on the third component, namely self-efficacy, measured through the SEMSA questionnaire seems to be more surprising. In fact, there is a tight relationship between self-efficacy and personality (Bandura, 1997), and it was also confirmed in the case of OSA syndrome and adherence to CPAP therapy in the long term (Maschauer et al., 2017). However, according to our results, the temperamental traits might not play a significant prominent role in self-efficacy when they are measured as a short-term outcome, as in our study. Future longitudinal studies should clarify this point. From a clinical perspective, our results might justify a multidisciplinary approach that does not take *a-priori* into account the temperamental traits of participants. This might be a piece of very crucial information because profiling the individual temperament through the questionnaires is a time-consuming activity. Such questionnaires, which consist of a very large number of items, might not be completely suitable in the case of individuals affected by OSA syndrome, who generally experience a higher level of mental fatigue, as well as attentional difficulties and a lower level of alertness (Llewellyn et al., 2008; Wilson et al., 2013; Alomri et al., 2021; Legault et al., 2021; Scarpina et al., 2021).

Some final considerations can be done about our study. The first one regards the scores registered at the temperament and Character Inventory-Revised (Martinotti et al., 2008), according to which our participants showed higher expressions of novelty seeking temperamental trait in comparison with the normative data (Martinotti et al., 2008), in agreement with recently published results provided by our group (Scarpina et al., 2021). We also reported that our participants showed higher expressions of the persistent temperamental trait. In this study, we enrolled individuals who participated and completed the residential four-week rehabilitation program. As reported by our group (Scarpina et al., 2021), this choice might be likely to be done by highly persistent individuals. As mentioned in the “Methods” section, a large percentage of our participants was affected by high-risk obesity, as well as OSA syndrome occurs frequently in obesity (Schwartz et al., 2008). In our residential treatment, participants were supported in increasing the amount of physical activity as well as in increasing their knowledge about good diet habits; in fact, we registered a significant decrease in BMI after the treatment. Weight reduction may be used as a secondary outcome in the treatment of OSA syndrome (Cayanan et al., 2019). Nevertheless, the benefits associated with CPAP therapy are not related to the baseline degree of obesity (Weaver et al., 2007). Previous studies investigating the temperamental traits according to Cloninger et al. (1993, 1994) in individuals affected by OSA syndrome with low-risk obesity reported heterogeneous results. Sforza et al. (2002) observed that individuals with OSA syndrome mostly showed higher expressions of novelty seeking temperamental traits in comparison with healthy individuals. This result was in agreement with the data reported in this study as well as in the study by Scarpina et al. (2021) relative to individuals with OSA syndrome and severe obesity. In contrast, Fìdan et al. (2013) registered no difference in the expression of the four temperamental traits between individuals affected by OSA syndrome and healthy individuals. Thus, we would suggest some cautions in interpreting our results, since the role of obesity and OSA syndrome on subjective psychological functioning is not clear-cut.

We concluded our discussion underlining the major limitation of our study, which was the absence of a control group of participants (i.e., individuals who did not follow the rehabilitation program). We described an observational study with a quasi-experimental pre–post design. However, since this study was done during hospitalization (which guarantees the treatments to every patient), it was not possible to randomize patients to a control group for ethical reasons (Bjarnason-Wehrens et al., 2007; Manzoni et al., 2011).

Understanding the primal beliefs and cognition of individuals about CPAP therapy in the case of OSA syndrome may provide insight into who might be likely to show a higher level of adherence. To summarize our results, the multidisciplinary residential four-week intervention described in this study seemed to positively change the level of risk perceptions, but not the components of perceived risks related to the disease and the self-efficacy about CPAP therapy. Nevertheless, the temperamental traits might play only a marginal role in the global changes reported by our participants after the intervention.

## Data Availability Statement

The raw data supporting the conclusions of this article will be made available by the authors, without undue reservation.

## Ethics Statement

The studies involving human participants were reviewed and approved by Istituto Auxologico Italiano. The patients/participants provided their written informed consent to participate in this study.

## Author Contributions

FS and AM conceived the study. FS supervised the entire study, performed the statistical analyses, and wrote the manuscript. IB and SC performed the data collection. LP, EG, IT, and MC selected the participants and performed the clinical assessment. GC and EM supervised the psychological assessment. PF and AM supervised the clinical assessment. All authors revised the manuscript.

## Conflict of Interest

The authors declare that the research was conducted in the absence of any commercial or financial relationships that could be construed as a potential conflict of interest.

## Publisher's Note

All claims expressed in this article are solely those of the authors and do not necessarily represent those of their affiliated organizations, or those of the publisher, the editors and the reviewers. Any product that may be evaluated in this article, or claim that may be made by its manufacturer, is not guaranteed or endorsed by the publisher.
