# Supplementary material for: Short-Term Effects of a Multidisciplinary Residential Rehabilitation Program on Perceived Risks, Confidence Toward Continuous Positive Airway Pressure Treatment, and Self-Efficacy in a Sample of Individuals Affected by Obstructive Sleep Apnea Syndrome
Source: Front Psychol. 2021 Aug 18;12:703089. doi: 10.3389/fpsyg.2021.703089 (PMC8416346; doi:10.3389/fpsyg.2021.703089)
Supplement: Supplementary file 1 [file Data_Sheet_1.pdf]

Supplementary Materials

Details relative to the participants excluded from the analyses.

As reported in the main text, fifty-two individuals (32 females; 20 males; mean age in years = 54.61; SD = 9.52; range = 34-75; mean education in years = 11.88; SD = 3.82; range = 5-23) were initially enrolled in the rehabilitation program; however, seven participants did not completed the rehabilitation program. In details, two participants were transferred in a different department of the hospital; one participant interrupted the treatment, since he/she was positive to COVID-19 infectious; finally, four participants resigned for the rehabilitative treatment. In Table S1, we report the scores relative to the collected measures for these four participants. Also, we compared their scores with those reported by the groups of participants who completed the rehabilitative treatments (reported in the main text), (Crawford, and Garthwaite, 2012).

Table S1. Scores at T0 relative to the four participants who resigned from the rehabilitation program are showed for all the assessed components. * when the score was significantly different from the included participants' mean.

	**#1**	**#2**	**#3**	**#4**
Gender	male	female	female	female
Age in years	40	53	48	73
Education in years	18	13	13	8
Body Mass Index	44.2	34.03	54.07	**24.97 ***
Mini Mental State Examination (score 0-30)	29	28	28	-
Clock Drawing Test (score 0-10)	10	10	9.5	-
Frontal Assessment Battery (score 0-18)	15	17	17	-

**SEMSA questionnaire**				
Perceived Risk (range score 1-4)	3	2	4	4
Outcome Expectancies (range score 1-4)	2	1	3	4
Treatment Self-Efficacy (range score 1-4)	3	2	1	4
**Temperament and Character Inventory-Revised**				
novelty seeking (range score 0-175)	**48 ***	**39 ***	**22 ***	**48***
harm avoidance (range score 0-165)	**41 ***	**36 ***	**42 ***	**33 ***
reward dependence (range score 0-150)	**58 ***	**61 ***	**47 ***	**75 ***
persistence (range score 0-175)	111	105	89	132
**Psychological well-being: Psychological General Well Being Index**				
anxiety (range score 0-25)	13	24	**1 ***	9
depression (range score 0-15)	15	15	**2 ***	10
positive well-being (range score 0-20)	10	17	**1 ***	6
self-control (range score 0-15)	12	14	7	8
general healthy (range score 0-15)	9	14	6	4
vitality (range score 0-20)	11	16	**2 ***	9
total score (range score 0-110)	70	100	**19 ***	46
**Sleep - quantitative measurement: polysomnography**				
Number of apnea/hypopnea events per hour of sleep (Apnea/Hypopnea Index - AHI)	68.8	14	84.4	15
Level of severity	severe	mild	severe	mild
**Sleep - subjective evaluation**				
Stanford Sleepiness Scale (range score 0-7)	2.66	1.38	2.55	2.33
Pittsburgh Sleep Quality Index (range score 0-21)	7	3	5	8
Epworth Sleepiness Scale (range score 0-24)	13	17	**23 ***	2

Flanker's Test				
Reaction time in milliseconds				
congruent	434	563	582	--
neutral	402	512	494	--
incongruent	439	557	563	--
no flanker	376	494	470	--
Percentage of accuracy				
congruent	92.5	92.5	57.5	--
neutral	92.5	95	95	--
incongruent	97.5	95	92.5	--
no flanker	90	97.5	95	--

These four participants reported lower scores relative to the temperamental traits of novelty seeking, harm avoidance and reward dependence, but similar level of persistent, when compared with the group of participants who completed the treatment. Crucially, subject #3 reported a significant lower level of quality of life, and specifically in the components of anxiety, depressive symptoms, positive well-being, and vitality. Finally, subject #4 reported a significant lower body mass index.

Supplementary Reference.

Crawford, J. R., & Garthwaite, P. H. (2012). Single-case research in neuropsychology: a comparison of five forms of t-test for comparing a case to controls. *Cortex*, *48*(8), 1009-1016.
